# Is food insecurity associated with dental caries?

**DOI:** 10.1038/s41432-023-00960-x

**Published:** 2024-01-05

**Authors:** Richard D. Holmes

**Affiliations:** Dental Public Health, School of Dental Sciences, Newcastle University, Newcastle upon Tyne, NE2 4BW UK

**Keywords:** Dental caries, Nutrition and diet in dentistry

## Abstract

**Data sources:**

Eight electronic databases including APA PsycINFO, CINAHL, Embase, LILACS, PubMed, Ovid, Scopus and Web of Science were searched from date of inception to November 2021. An updated search was conducted in August 2022. Google Scholar was accessed including Open Grey and ProQuest. Reference lists of the included studies were analysed for potentially eligible studies.

**Study selection:**

Observational studies (cross-sectional, case-control and cohort) that evaluated the association between dental caries and food insecurity were eligible for analysis. Qualitative studies, reviews and meeting abstracts were excluded. There were no restrictions on language or publication date.

**Data extraction and synthesis:**

Two reviewers independently screened titles and abstracts. A third experienced researcher was consulted if there was disagreement. Food insecurity status was the exposure with dental caries the outcome. The authors retrieved effect measures, 95% CI’s and *P* values where available. Heterogeneity was assessed via I^2^ and R^2^. A total of 514 records were initially identified. Once duplicates were removed, 19 references were assessed in full. The association between food insecurity and dental caries were presented as odds-ratios, relative risks and prevalence ratios with 95% CIs. A random-effects model was fitted to all meta-analyses.

**Results:**

Evaluation identified 14 studies for the qualitative synthesis and 7 studies for the quantitative synthesis. The total sample size for the 14 studies was 150,546 individuals. Quantitative data merged from two studies found food-insecure individuals more prone to dental caries than food-secure individuals (OR = 1.62; 95% CI, 1.01–2.60; *P* = 0.045). In two studies that used binary data to compare food security or insecurity, food insecure individuals were more likely to exhibit dental caries (OR = 1.66; 95% CI, 1.36–2.02; *P* < 0.0001).

**Conclusions:**

People experiencing food insecurity are more likely to exhibit dental caries than those who have food security.

## A Commentary on

**Drumond V Z, de Arruda J A, Bernabé E, Mesquita R A, Abreu L G**.

Burden of dental caries in individuals experiencing food insecurity: a systematic review and meta-analysis. *Nutr Rev* 2023; **81:** 1525–1555.

**GRADE Rating**: 

## Commentary

The 2030 Agenda for Sustainable Development incorporates 17 Sustainable Development Goals (SDGs) which include zero hunger, good health and well-being, quality education, reduced inequalities, and partnerships for the goals^1^. The topic area for this systematic review and meta-analysis touches upon several areas of major public health concern for policymakers globally.

The research question was: ‘Are individuals in a status of food insecurity more likely to exhibit dental caries than individuals in a status of food security?’. A systematic review and meta-analysis were appropriate methods to answer this. A strength of the study was that it was registered on the PROSPERO database^2^ and it adhered to MOOSE guidelines (Meta-analysis Of Observational Studies in Epidemiology) which help to ensure a standardised approach to the reporting of meta-analyses^3^.

Clear eligibility criteria were stated with regard to the included observational studies. However, as temporality cannot be established using these study designs, it is not possible to infer causality. There is also a possibility that some participants from the included studies may have developed dental caries prior to being exposed to conditions leading to food insecurity. The PROSPERO record reported that individuals of any age, sex and ethnicity were eligible.

Exposure status (food insecurity) used the Household Food Security Survey Measure^4^ or the Brazilian or Korean versions. However, in two studies, the method used to assess food insecurity was either not described or it was measured by a questionnaire developed by researchers. There was variation across the primary studies relating to how outcomes (dental caries) were recorded, but it was reported that 13 studies used reliable clinical examination methods with trained clinicians. Six studies used DMFT or dmft, 5 studies adopted a simple dichotomous assessment (presence or absence of dental caries) and in 2 studies dental caries was recorded by patient self-report measures.

A strength of this systematic review and meta-analysis is that a sizable number of participants were included (150,546 individuals); however, these individuals were drawn from a small number of studies (*n* = 14). A real limitation of the review’s findings is that individuals from only three countries (United States, Brazil and South Korea) were represented in the included studies. It is not surprising, therefore, that evaluation revealed serious concerns with respect to indirectness according to the GRADE approach^5^. An example of indirectness is where study participants differ from those of interest. This limits the potential applicability of the findings and suggests a need for future research studies to include a more diverse range of countries including ‘low’ and ‘low-middle’ economies (by income) as classified by the World Bank^6^.

Risk of bias was assessed across relevant domains with most primary studies achieving a satisfactory outcome. The GRADE tool enabled the authors to report no concerns related to publication bias in the meta-analyses. However, only three of the included studies reported a response rate and none of the 14 included studies provided any information about the comparability between respondents and non-respondents. The strength of the evidence for meta-analysis of binary data was rated as ‘very low’ and for the inverse-variance meta-analyses the strength of the evidence was ‘low’.

The findings from this review highlight the need for decision-makers to continue developing healthy public policy for all and particularly for those from low-income and food-insecure households. The authors of this systematic review and meta-analysis identified that in order to explore a potential causal relationship between dental caries and food insecurity, longitudinal studies are required. Good-quality and well-conducted longitudinal studies would provide a sensible focus for future research in this area.

Practice points

- Oral health professionals have an important role to play in multidisciplinary health care teams, particularly those linked to vulnerable individuals affected by food insecurity.
- Policymakers should focus upon low-income urban and rural locations to address local infrastructure issues pertaining to low food access, the availability of a wide variety of foodstuffs and the affordability of healthy food.
